# Impact of digital clinical decision support on quality of care and antibiotic stewardship for children under five in South-Central Somalia

**DOI:** 10.1093/oodh/oqae029

**Published:** 2024-12-02

**Authors:** Eveline Hürlimann, Marco Landi, Alli Miikkulainen, Camille Renner, Capucine Musard, Hassan Hussein Mohamed, Hassan Abdullahi Ali, Omar Sheik Mohamud, Abdifatah Ahmed Mohamed, Talia Salzmann, Fenella Beynon, Anja Junker

**Affiliations:** Department of Medical Parasitology and Infection Biology, Swiss Tropical and Public Health Institute (Swiss TPH), Kreuzstrasse 2, 4123 Allschwil, Switzerland; Faculty of Science, University of Basel, Klingelbergstrasse 50, 4056 Basel, Switzerland; Department of Primary Health Care, Somalia Delegation in Kenya (SOK), International Committee of the Red Cross (ICRC), Ngecha Road, Off Lower Kabete Road, P.O. Box 73226 - 00200 – Nairobi, Kenya; Department of Primary Health Care, Somalia Delegation in Kenya (SOK), International Committee of the Red Cross (ICRC), Ngecha Road, Off Lower Kabete Road, P.O. Box 73226 - 00200 – Nairobi, Kenya; Faculty of Science, University of Basel, Klingelbergstrasse 50, 4056 Basel, Switzerland; Swiss Center for International Health, Swiss Tropical and Public Health Institute (Swiss TPH), Kreuzstrasse 2, 4123 Allschwil, Switzerland; Faculty of Science, University of Basel, Klingelbergstrasse 50, 4056 Basel, Switzerland; Swiss Center for International Health, Swiss Tropical and Public Health Institute (Swiss TPH), Kreuzstrasse 2, 4123 Allschwil, Switzerland; Department of Primary Health Care, Somalia Delegation in Kenya (SOK), International Committee of the Red Cross (ICRC), Ngecha Road, Off Lower Kabete Road, P.O. Box 73226 - 00200 – Nairobi, Kenya; Department of Primary Health Care, Somalia Delegation in Kenya (SOK), International Committee of the Red Cross (ICRC), Ngecha Road, Off Lower Kabete Road, P.O. Box 73226 - 00200 – Nairobi, Kenya; Department of Primary Health Care, Somalia Red Crescent Society (SRCS), Afgoi Road, Sope 5km, Wadajir District, Mogadishu, Somalia; Department of Policy and Planning, Federal Government of Somalia, Ministry of Health & Human Service, Bondhere District, Mogadishu, Somalia; Faculty of Science, University of Basel, Klingelbergstrasse 50, 4056 Basel, Switzerland; Swiss Center for International Health, Swiss Tropical and Public Health Institute (Swiss TPH), Kreuzstrasse 2, 4123 Allschwil, Switzerland; Faculty of Science, University of Basel, Klingelbergstrasse 50, 4056 Basel, Switzerland; Swiss Center for International Health, Swiss Tropical and Public Health Institute (Swiss TPH), Kreuzstrasse 2, 4123 Allschwil, Switzerland; Faculty of Science, University of Basel, Klingelbergstrasse 50, 4056 Basel, Switzerland; Swiss Center for International Health, Swiss Tropical and Public Health Institute (Swiss TPH), Kreuzstrasse 2, 4123 Allschwil, Switzerland

**Keywords:** clinical decision support system (cdss), primary health care, integrated management of childhood illness (imci), child health, Somalia, antimicrobial stewardship, digital health

## Abstract

In the context of protracted conflict, severe droughts and health system constraints, children under-five in Somalia face one of the highest mortality rates in the world. The WHO Integrated Management of Childhood Illness (IMCI) guidance targets the main causes of morbidity and mortality, but adherence is low. We implemented the ALgorithm for the MANAgement of CHildhood illness (ALMANACH), a digital clinical decision support system, with the aim of improving IMCI adherence whilst promoting antibiotic stewardship in South-Central Somalia. Alongside, we evaluated health service delivery and ALMANACH acceptability and impact to inform design and roll-out. A pre-post assessment involving direct observation of consultations with sick children (2–59 months) based on the Demographic and Health Surveys Service Provision Assessment, complemented by exit interviews with caregivers and feedback from healthcare staff and stakeholders. Over 600 consultations were observed in each assessment period, in seven health facilities. ALMANACH had a significant impact on antibiotic prescription (reduction from 58.1% pre- to 16.0% post-implementation). This was particularly pronounced among certain conditions such as upper respiratory tract infections (30-fold reduction, RR = 0.03). Large differences in guideline adherence were observed (danger signs: 1.3% pre- to 99% post-implementation; counselling on follow-up: 12% pre- to 94% post-; and Vitamin A supplementation need checked: 19.9% pre- to 96.1% post-implementation). ALMANACH was found to be acceptable to caregivers, healthcare providers and stakeholders, with reports of positive impact on perceived quality of care. Implementation of ALMANACH in primary healthcare in Somalia significantly improved quality of care and guideline adherence, supporting the use of ALMANACH and similar tools to improve healthcare in fragile and resource-constrained settings.

**RESUMEN:**

En un contexto de conflicto prolongado, sequías severas, y limitaciones en el sistema de salud, los niños menores de 5 años en Somalia sufren una de las tasas de mortalidad más altas del mundo. La estrategia Atención Integrada a las Enfermedades Prevalentes de la Infancia (AIEPI) de la OMS incluye recomendaciones alrededor de las causas principales de morbilidad y mortalidad, pero la adherencia a esta guía es pobre. Implementamos el algoritmo para la gestión de enfermedades de la infancia ALMANACH (ALgorithm for the MANAgement of CHildhood illness), un sistema digital de apoyo para las decisiones clínicas, a fin de mejorar el cumplimiento de la AIEPI durante un esfuerzo de promoción de la correcta administración de antibióticos en el centro-sur de Somalia. De manera paralela, evaluamos la prestación de servicios de salud, y la aceptabilidad e impacto de ALMANACH, para informar su diseño y lanzamiento. Evaluación antes-después de la implementación del algoritmo, derivada de la observación directa de consultas médicas para niños enfermos (de 2 a 59 meses), basada en la Evaluación de Provisión de Servicios (SPA, por sus siglas en inglés) de DHS (Demographic and Health Surveys, Encuestas Demográficas y de Salud), complementada con encuestas de salida a los cuidadores, y retroalimentación del personal de salud y partes interesadas. Se observaron más de 600 consultas en cada periodo de evaluación, en 7 instalaciones de salud. ALMANACH mostró tener un impacto significativo en la prescripción de antibióticos (con una reducción de 58.1% antes de la implementación, a 16.0% después). Esto fue particularmente pronunciado con ciertas condiciones, como las infecciones de vías respiratorias superiores (ocurriendo 30 veces menos, RR = 0.03). Se observaron grandes cambios en la adherencia a las recomendaciones (atención a signos de peligro: de 1.3% antes de la implementación, a 99% después; orientación acerca del seguimiento: de 12%, antes, a 94% después; y prueba de necesidad de vitamina A suplementaria: de 19.9%, antes, a 96.1% después). El ALMANACH le resultó aceptable a los cuidadores, al personal de salud y a las partes interesadas, con reportes de impacto positivo en la calidad percibida del cuidado. La implementación de ALMANACH en la atención primaria de salud en Somalia resultó en una calidad de cuidados y adherencia a las recomendaciones significativamente mayores, favoreciendo el uso de ALMANACH y herramientas semejantes en el mejoramiento del cuidado de la salud en entornos frágiles y de recursos limitados.

**RESUMO:**

No contexto de conflitos prolongados, secas graves e limitações do sistema de saúde, as crianças com menos de cinco anos na Somália enfrentam uma das taxas de mortalidade mais elevadas do mundo. As orientações da OMS sobre a Gestão Integrada das Doenças da Infância (GIDI) visam as principais causas de morbilidade e mortalidade, mas a adesão é baixa. Implementámos o ALgorithm for the MANAgement of CHildhood illness (ALMANACH), um sistema digital de apoio à decisão clínica, com o objetivo de melhorar a adesão à IMCI, promovendo simultaneamente a gestão de antibióticos no centro-sul da Somália. Paralelamente, avaliámos a prestação de serviços de saúde, e a aceitabilidade e o impacto do ALMANACH para informar a sua conceção e implementação. Uma pré/pós-avaliação que envolveu a observação direta de consultas com crianças doentes (2–59 meses) com base na Avaliação da Prestação de Serviços do DHS, complementada por entrevistas à saída com os prestadores de cuidados e feedback dos profissionais de saúde e das partes interessadas. Foram observadas mais de 600 consultas em cada período de avaliação, em 7 unidades de saúde. O ALMANACH teve um impacto significativo na prescrição de antibióticos (redução de 58,1% antes da implementação para 16,0% após a implementação). Este impacto foi particularmente pronunciado em determinadas doenças, como as infeções do trato respiratório superior (redução de 30 vezes, RR = 0,03). Foram observadas grandes diferenças na adesão às directrizes (sinais de perigo: 1,3% antes da implementação para 99% após a implementação; aconselhamento no seguimento: 12% antes para 94% depois; e necessidade de controlo da suplementação com vitamina A: 19,9% antes da implementação para 96,1% após a implementação. O ALMANACH foi considerado aceitável pelos cuidadores, prestadores de cuidados de saúde e partes interessadas, com relatos de um impacto positivo na perceção da qualidade dos cuidados. A implementação do ALMANACH nos cuidados de saúde primários na Somália melhorou significativamente a qualidade dos cuidados e a adesão às directrizes, apoiando a utilização do ALMANACH e de ferramentas semelhantes para melhorar os cuidados de saúde em contextos frágeis e com recursos limitados.

**RÉSUMÉ:**

Dans le contexte d’un conflit prolongé, de graves sécheresses et de contraintes du système de santé, les enfants de moins de cinq ans en Somalie sont confrontés à l’un des taux de mortalité les plus élevés au monde. Les lignes directrices de l’OMS sur la prise en charge intégrée des maladies de l’enfant (PCIME) ciblent les principales causes de morbidité et de mortalité, mais leur observance est faible. Nous avons mis en œuvre ALgorithm for the MANAgement of CHildhood illness (ALMANACH), un système numérique d’aide à la décision clinique, dans le but d’améliorer l’observance à la PCIME tout en promouvant la gestion responsable des antibiotiques dans le centre-sud de la Somalie. Parallèlement, nous avons évalué la prestation de services de santé, ainsi que l’acceptabilité et l’impact d’ALMANACH pour éclairer la conception et le déploiement. Une évaluation pré-post impliquant l’observation directe des consultations des enfants malades (2–59 mois) basée sur l’Évaluation des prestations de services de l’EDS, complétée par des entretiens de sortie avec les soignants et les commentaires du personnel de santé et des parties prenantes. Plus de 600 consultations ont été observées au cours de chaque période d’évaluation, dans 7 formations sanitaires. ALMANACH a eu un impact significatif sur la prescription d’antibiotiques (réduction de 58,1% avant la mise en œuvre à 16,0% après la mise en œuvre). Cela était particulièrement prononcé dans certaines affections telles que les infections des voies respiratoires supérieures (réduction de 30 fois, RR = 0,03). De grandes différences dans le respect des lignes directrices ont été observées (signes de danger: 1,3% avant à 99% après la mise en œuvre; conseils sur le suivi: 12% avant à 94% après la mise en œuvre; et vérification du besoin de supplémentation en vitamine A: 19,9% avant 96,1% après la mise en œuvre). ALMANACH s’est avéré acceptable pour les soignants, les prestataires de soins de santé et les parties prenantes, avec des rapports faisant état d’un impact positif sur la qualité perçue des soins. La mise en œuvre d’ALMANACH dans les soins de santé primaires en Somalie a considérablement amélioré la qualité des soins et le respect des lignes directrices, encourageant l’utilisation d’ALMANACH et d’outils similaires pour améliorer les soins de santé dans des contextes fragiles et aux ressources limitées.

## INTRODUCTION

Somalia, the most eastern country in Africa, faces many challenges. The protracted armed conflict and recent unprecedented droughts [1] have impacted lives and livelihoods in dramatic ways, leaving more than 8.3 million people in urgent need of humanitarian assistance [2]. Children under 5 years of age are particularly vulnerable, and the under-five mortality ratio in Somalia remains among the highest in the world. One of every eight Somali children dies before their 5th birthday (112 deaths/1000 live births) [3] and the vast majority of these deaths are from preventable causes—pneumonia, diarrhea, measles and malnutrition and neonatal disorders [4].

The Integrated Management of Childhood Illness (IMCI) strategy, first developed by the World Health Organization (WHO) and United Nations Children’s Fund (UNICEF) three decades ago, was designed specifically to address these main causes of under-five morbidity and mortality in low-resource settings. Since inception, IMCI has been implemented in numerous countries with demonstrated important benefits to child health [5]. However, its full potential is often not exploited due to low adherence by healthcare workers to the evidence-based recommendations for managing sick children at the core of the IMCI strategy [6–9].

Adherence to clinical guidance such as IMCI, and many other aspects of health service provision, face major barriers in Somalia. The continuing conflict and absence of effective central health system governance have led to the breakdown of health infrastructure and prevented institutional investment in health services [10]. The Ministry of Health (MoH) is funded almost exclusively by external donors, while hundreds of private practitioners are operating in clinics, hospitals, pharmacies and shops, in a largely deregulated system [11]. Though over 600 facilities are listed in the MoH platform, most are unable to provide services consistently due to recurrent funding gaps [4].

The health workforce crisis exacerbates the situation—Somalia has one of the lowest health workforce densities globally (0.11 per 1000 population, 40 times lower than the sustainable development goal index threshold of 4.45), and health worker (HW) skills have been limited by a lack of training and supervision opportunities [12]. The challenge of ‘under-provision’ of health services is compounded by inappropriately high use of antibiotics. Globally, overuse of antibiotics disproportionately affects children, with an estimated 18.5–25 courses of antibiotics being prescribed in a child’s first 5 years of life in low- and middle-income countries [13, 14]. Antimicrobial resistance has been recognized as one of the biggest public health challenges in Somalia and represents a priority for the MoH [15–17]. But when faced with high under-five infection-related morbidity and mortality such as that in Somalia, efforts to reduce antibiotic prescribing need to be paired with broader efforts to improve quality of care.

Clinical decision support systems (CDSS) have shown potential to contribute to addressing these challenges. By providing recommendations to health workers tailored to individual patient information based on an underlying clinical algorithm, CDSS can improve adherence to clinical guidelines and as such have been recommended by WHO to support health systems strengthening [18]. Several IMCI-based CDSS have been developed and implemented in resource-constrained settings [19–22]. However, while improvements in quality of care and clinical outcomes have been demonstrated, there is wide variation in effectiveness seen [23–28]. Given the complex nature of such interventions, this may be explained by a combination of the diversity in context, design and implementation approach of IMCI-based CDSS [21]. Systematic reviews and qualitative syntheses of broader literature on CDSS and other mobile health tools have highlighted the substantial heterogeneity in effectiveness and acceptability of such interventions, and called for more evidence to help address gaps in understanding on how to optimize impact [29, 30].

It is in this context that we present the findings from the evaluation of the implementation of the ALgorithm for the MANAgement of CHildhood illness (ALMANACH), an IMCI-based CDSS for children 2–59 months of age in South-Central Somalia. The overarching aim of the intervention was to improve adherence to IMCI and strengthening quality of care and antimicrobial stewardship. Alongside implementation of ALMANACH, monitoring and evaluation activities were conducted with the objectives of:

- Understanding service delivery and HW perspectives prior to ALMANACH implementation to inform initial intervention design
- Evaluating the impact of ALMANACH on clinical service provision, including antimicrobial stewardship, to inform scale-up
- Determining the acceptability of ALMANACH to HWs and caregivers to inform refinements to the CDSS and broader implementation package, including social mobilization, information, education and communication strategies

This paper describes the results of these evaluation activities with the aim of sharing learning on the implementation and impact of IMCI-based CDSS in South-Central Somalia where, to our knowledge, no other facility-based CDSS have been implemented for this particularly vulnerable group of children under 5 years of age.

## METHODS

### Evaluation design

The evaluation was comprised of three main components: a pre-post assessment of clinical care involving direct observations of consultations conducted by HWs with sick children 2–59 months of age to inform the initial design and evaluate impact of ALMANACH on guideline adherence and antibiotic prescription; post-implementation structured exit interviews with caregivers of sick children to understand intervention acceptability and understanding of care provided; and feedback collected from HWs, caregivers and other stakeholders through group discussions and engagement sessions to inform intervention design and understand acceptability.

### Setting

Implementation and evaluation of ALMANACH were nested within the broader activities of the Somali Red Crescent Society (SRCS) and International Committee of the Red Cross (ICRC) in South-Central Somalia. At the time of the study SRCS managed 30 Primary Health Care (PHC) facilities with support from the ICRC on management, monitoring, infrastructure and medical supply. South-Central Somalia was the region most heavily affected by conflict, resulting in an extremely challenging operational context given the volatile and constraining security situation. Severe droughts and outbreaks of cholera and measles were common, and the region had the highest under-five mortality in the country. The facilities included in the evaluation had a total catchment population of 142 096 (ranging from 11 000 to 32 000 per facility), of which 26 998 were children under-5 (ranging from 2000 to 6000 per facility); detail is provided in supplementary Table S1. Facilities were far away from each other and from the secondary level of care, which rendered successful referrals of patients difficult. The usual opening hours for the clinics of SRCS were from 7:30 AM up to 2 PM; and on distribution days of the outpatient therapeutic program for patients with severe acute malnutrition (SAM), clinics could be crowded with up to 100 children. Services for sick children 2–59 months of age were generally provided by nurses who had received IMCI training. Refresher training was generally provided every 1–2 years, with one occurring during our assessment period in September 2021. A printed version of the WHO IMCI booklet was available in the consultation room in all facilities prior to the pre-intervention period and any ALMANACH activities. Health services were free of charge for patients, including consultations fees and medical consumables.

**Figure 1:**
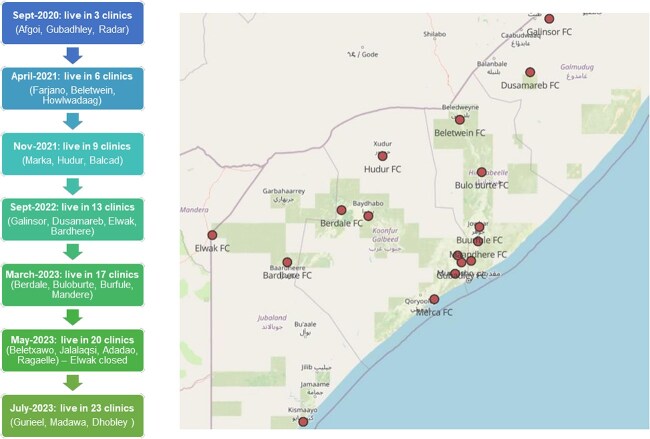
Implementation phases and facilities of ALMANACH Somalia

### Intervention

The intervention encompassed the tablet-based ALMANACH CDSS, training, supervision and HW and community engagement.

The first version of ALMANACH was originally developed in a research context by the Swiss Tropical and Public Health Institute (Swiss TPH) over ten years ago and evaluated in partnership with the Ifakara Health Institute in Tanzania [31]. Subsequently, through partnership between Swiss TPH, the ICRC and local partners, including MoH and other actors, it has since then been updated, adapted, implemented and evaluated in Nigeria and Afghanistan [23, 24, 27], and is in the early stages of implementation in Libya.

The development of ALMANACH Somalia began in late 2019 through a partnership between the SRCS, ICRC and Swiss TPH. The underlying clinical decision algorithm (CDA) was informed by existing templates [27, 31], further developed and adapted to fit the Somalian context. To do so, national and international guidelines, local epidemiological considerations (most common clinical presentations), diagnostic and treatment capacity, and standard operating procedures were reviewed and applicable modifications included. The ‘human-readable’ algorithm was designed using Microsoft Visio 2016 and validated by expert review panels consisting of clinicians from ICRC, SRCS, Médecins Sans Frontières, Swiss TPH and external independent experts.

The algorithm was then translated into an electronic format using CommCare (Dimagi Inc., Cambridge, USA) for use by HWs as the ALMANACH app on android tablets and validated against clinical test cases before use in clinics. After final testing in English, the algorithm was translated into Somali language, validated and again tested. The algorithm could be displayed in either language and HWs could change language setting within one consultation. The algorithm could be used offline; periodic internet connection was only required for synchronization of electronic data captured during consultations. Data (which contained no names or dates of birth) were stored on the CommCare server. They were then transferred via the OpenHIM mediator (available on Github: https://github.com/SwissTPH/openfn-openhim-mediator) and aggregated for display in a dashboard for project partners on the Swiss TPH hosted DHIS2 server.

Prior to any field activities, group discussions were held with all HWs involved in ALMANACH as well as their respective supervisors, branch health officers. The discussions introduced the purpose and features of ALMANACH, and explored expectations, concerns and challenges ahead of implementation. Engagement and sensitization campaigns took place with all relevant stakeholders in the anticipated intervention areas. This included discussions with the local health authorities, health promoters, female community HWs and community health committees (CHCs), using pre-designed communication materials by SRCS and ICRC which told a story about a Somali child in need of medical care. Engagement and sensitization continued as a regular activity following ALMANACH introduction to encourage health seeking behavior.

Before using ALMANACH independently, HWs received a 4-day training on ALMANACH, focused on the use of the application and safe prescribing. This classroom-based training was followed by a 2-day on-the-job training with practical use of ALMANACH during consultations with sick children. Close supervision and additional support during in-service training continued through visits and regular interaction with the project team. Brief bulletins with a summary from the dashboards, along with messages of the month from feedback captured (with consent) from HWs, caregivers and other project stakeholders were shared on a monthly basis with CHCs and internally with SRCS, ICRC and Swiss TPH.

Implementation took place in a phased approach, starting in September 2020 (Fig. 1), for logistical reasons and to enable learning and refinement of the CDA and overall implementation approach. For each update to the algorithm, the same approach was followed as described earlier (updates to the drawn algorithm, expert panel review, translation to CommCare, and testing).

### Pre-post assessment of clinical care

**Figure 2:**
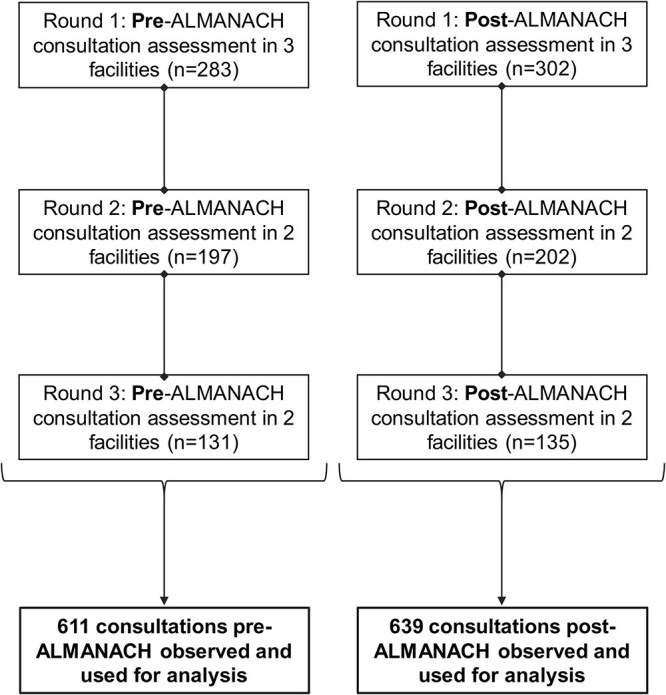
Flow chart of the study. Number of consultations observed by assessment time point

Direct observations of clinical consultations were conducted in a total of seven ICRC-supported SRCS-managed health facilities in the South-Central Somali region between February 2021 and July 2022. Health facilities were visited shortly before implementation and then re-visited within a maximum of 3 months after implementation of ALMANACH. A phased approach was taken to align with implementation activities and to acknowledge the availability of two qualified observers only for the assessments. Facilities were visited in three different rounds; three facilities were evaluated first, two in the second round and another two in the third round (see Fig. 2). The facilities assessed were situated in Houlwadaag, Farjano, Beletweyn, Hudur, Balat, Dusamareb and Galinsor. An additional three facilities originally planned to be part of the assessment were not included due to security concerns.

Consultations were considered eligible for observation if the child was between 2 to 59 months of age and attending for an illness, and the caregiver provided consent. Agreement was also sought from the HW. Practically, this was implemented by screening and enrolling at the under-five consultation room (sick children), and the observer leaving if the child was under 2 months of age or the caregiver did not provide consent.

Given the intention of the pre-post assessment to both provide a broad indication of service delivery to inform intervention design and refinement, and to assess the impact of introducing ALMANACH on antibiotic prescription and key indicators of adherence to IMCI, we based the assessment on the sick child observation of the validated Demographic and Health Surveys (DHS) Service Provision Assessment (SPA) approach [32] which focused on quality and content of service delivery.

In addition to gathering broad information on IMCI adherence, specific outcomes were selected based on consultation with stakeholders on local priorities:

Primary outcome:

- Proportion of consultations with an antibiotic prescription, expected to reduce as a result of ALMANACH introduction

Secondary outcomes:

- Proportion of children with upper respiratory tract infections (URTIs) inappropriately receiving antibiotic treatment, expected to reduce as a result of ALMANACH introduction and used as a proxy for appropriate treatment
- Proportion of consultations with a parasitosis classification, expected to reduce as a result of ALMANACH introduction, used as a proxy for appropriate diagnosis as parasitosis is almost always an inappropriate diagnosis/classification in the absence of microscopy
- Proportion of consultations in which the need for Vitamin A supplementation was checked, expected to increase as a result of ALMANACH, as a proxy for better adherence to IMCI prevention measures

As additional indicators for improved quality of care, we evaluated proportions of children assessed for general danger signs, main symptoms, clinical signs and tests where relevant as indicated by IMCI/ALMANACH (e.g. pallor for all children, malaria RDT for children with fever), classification of main IMCI conditions, appropriate treatment (e.g. oral rehydration solution [ORS] and zinc for acute watery diarrhea). In addition, we assessed consultation duration.

The sample size was originally estimated based on being able to detect a difference of 10% in the primary outcome of antibiotic prescriptions from an anticipated baseline of 50% (estimated from facility register data), with a power of 0.8 and an error of 0.05, resulting in a need to observe an estimated 388 consultations for each time point (pre-post) in each of the three rounds. Due to logistical considerations (security, the constraints imposed as a result of the SARS-CoV2 pandemic, and the importance of retaining focus on implementation/operations), and as a result of observing a more marked reduction in antibiotic prescriptions when initially piloting the observation tool (30%, from a baseline of 66%), we prioritized questions of feasibility and opted for a pragmatic sample size of ~100 consultations per health facility per time point.

The data collection tool, adapted from the DHS SPA sick child observation tool, was first developed on paper and tested during a pilot assessment between June and November 2020 in three health facilities (Radaar, Gubadhley and Afgoye), after which it was further adapted and converted into an electronic data capture format using Open Data Kit (ODK). In addition to piloting the tool and processes, the data collected was used to understand service provision in routine practice to guide implementation decisions, but is not part of the analysis presented in this article.

After the first round of pre-post assessments included in the evaluation, further feedback was gathered from the data collectors about usability of the ODK questionnaire and potential areas of improvement. As a consequence, the questionnaire was slightly adapted to facilitate data entry (e.g. by adding more answer options or extending them by using generic terms and improved display for data input). Its structure and the questions remained the same as in the previous version of the questionnaire to ensure comparability with subsequent phases of the study.

Observations were conducted by one of two observers, both being originally trained as nurses and acting as health field officers supporting the implementation of ALMANACH. The observers were initially trained in detail on the content and correct practices of the questionnaire used during observations, as well as on the concept of good clinical practice. Prior to each new assessment at pre- or post-ALMANACH, a refresher training was provided by the evaluation team. Daily data cleaning was performed to ensure consistency and quality of data collected during each assessment.

During the observations done in the three first facilities (round 1, pre-implementation), both observers conducted assessments concurrently in the facilities to share experience at the end of the day and ensure consistency in approach to applying the questionnaire. The first day of observations (pre- and post-implementation) in the first round of assessment were considered as training day and data was not included for analysis. Each assessment round took between 4 and 7 days per facility per time point.

Following informed consent, data was gathered by observing the consultations and collecting all information directly into the tablet-based ODK questionnaire. HWs were asked to think out loud and comment on their actions to facilitate the assessment. In case of serious errors that could endanger the patient, observers were advised to intervene in line with WHO guidance [33]. Once the consultation was completed and the patient had left the room, the observer collected information from written documentation (e.g. patient registry, medicine prescriptions) if needed to complement the data collected. No personally identifiable information was collected. Data was stored on ODK central and quality checks were performed regularly by the evaluation team.

For the indicators representing a proxy for the provided quality of care together with overall antibiotic prescriptions, we calculated risk ratios (RRs) and 95% confidence intervals (CIs) to determine the extent of difference between baseline and post-ALMANACH implementation (effect size). In a next step, we built logistic regression models that allowed for adjustment of potentially influencing covariates including age group, sex, facility, season, observer, additional refresher IMCI training received during the period of assessment, fever (present and reported) or parasitosis as diagnosis. All remaining parameters and indicators were analyzed descriptively, compared between pre- and post-implementation and presented in frequency tables. Data were analyzed using Stata version 16.

### Exit interviews with caregivers of sick children

Exit interviews were conducted, in the same seven facilities as the pre-post evaluation, to explore the acceptability of ALMANACH to caregivers, and capture their understanding of care provided. About 50 exit interviews per facility were conducted, with the assessment conducted in two stages, in June and December 2022, completely independently from the pre-post study. The process and tool were tested in one facility prior to the main data collection activities.

Following informed consent, the questionnaire was administered by trained interviewers, independent to the project, to eligible caregivers (of children 2–59 months of age attending the under-five clinic) after their consultation but prior to leaving the facility. The structured questionnaire contained a minimum of 13 and maximum of 23 items, depending on responses, covering services provided (e.g. diagnoses, treatments and/or counselling provided and explained; the tablet being used) awareness and satisfaction (e.g. confidence in and prior knowledge of the tool; medication expectation; waiting time). Data were entered by the interviewer directly onto a tablet-based ODK form. No personally identifiable information was captured. Descriptive analysis was performed using Microsoft Excel.

### Feedback from group discussions and engagement sessions

Feedback to inform the intervention design and understand acceptability was obtained through a range of engagement activities both prior to and during implementation. Pre-intervention, group discussions were held with HWs, supervisors and branch health officers (outlined further under ‘intervention’). Separately, discussions were held with local health authorities, health promoters, female community HWs and the CHC. Notes were recorded by facilitating members of the SRCS project team, which were then synthesized and reviewed with the wider SRCS, ICRC and Swiss TPH project team.

Following implementation, regular visits to health facilities and further engagement with health authorities, community HWs and the CHC were made by members of the project team. Following verbal consent, project staff asked for feedback (both positive and negative) to understand acceptability and inform intervention adaptation. Similarly, notes were taken, transcribed and synthesized for review by the project team, with samples selected for each monthly bulletin.

### Ethical considerations

The evaluation activities were approved by the Ethical review Board of ICRC, the leadership of SRCS, and the Federal MoH. Prior to the clinical observations, all caregivers of children to be seen by the HW were asked to provide informed written or oral consent to allow the observer to attend their consultation. In view of high illiteracy rates, the implementation team invested in substantial information and sensitization sessions in the communities prior to the observations. The same procedure was applied for caregivers invited to participate in exit interviews after consultations. Verbal informed consent was obtained to take notes and use quotes from group discussions and other engagement sessions with HWs, caregivers and other key stakeholders. No personally identifiable information was collected for any of the evaluation activities.

## RESULTS

From the start of implementation in September 2020, a total of 187 873 consultations have been performed using ALMANACH, of which 4508 were in 2020, 35 533 in 2021, 69 093 in 2022, and 78 739 up to the end of October 2023. With the phased approach to roll-out, this equated to an average of around 500 consultations per month per facility.

### Pre-post evaluation of clinical care

A total of 1250 (99.9%) caregivers of sick children 2–59 months of age provided consent, of which 1001 (80.1%) were provided verbally. Observations were conducted for 611 consultations pre-implementation and 639 consultations post-implementation, over a total of 76 observation days (38 in each period), as shown in Fig. 2. There were slightly fewer consultations with girls 295 (48.3%) pre- and 315 (49.3%) post-implementation. More than half of consultations observed 354 (57.9%) pre- and 378 (59.2%) post-ALMANACH were with children under the age of 2.

All 611 (100%) consultations pre-ALMANACH and 636 (99.5%) post-ALMANACH were done by under-five nurses; the remaining three post-implementation consultations were done by a replacement nurse that received prior training on the tool. HWs used ALMANACH in all 639 post-implementation consultations. The median time needed for a consultation was 4 min and 18 s pre-implementation and 7 min and 12 s after ALMANACH implementation.

#### Primary and secondary outcomes

Following introduction of ALMANACH in the health facilities, children consulted were nearly 4 times (RR = 0.27, 95% CI = 0.23–0.33) less likely to receive an antibiotic treatment (Table 1).

**Table 1:** Significant effects on proxies for quality of care assessed in 611 and 639 patients pre- and post-ALMANACH implementation, respectively

**Impact**	**Proxy**	**Pre-implementation n/N (%)**	**Post-implementation n/N (%)**	**Effect size**			
				**RR**	**95% CI RR**	**aOR** ^**d**^	**95% CI**
*Improved treatment*							
	Antibiotic prescriptions overall	355/611 (58.1%)	102/639 (16.0%)	0.27	0.23–0.33	0.14	0.09–0.23
	Antibiotic prescriptions excluding intestinal parasitosis^a^	246/478 (51.5%)	102/639 (16.0%)	0.31	0.25–0.38	0.13	0.08–0.22
	Inappropriate antibiotic prescriptions for patients diagnosed with URTI^b^	112/116 (96.6%)	1/32 (3.1%)	0.03	0.01–0.22	NA	NA
*Improved diagnosis*							
	Inappropriate classification as parasitosis	135/611 (22.1%)	0/639 (0.0%)	NA	NA	NA	NA
*Improved prevention*							
	Vitamin A need checked in eligible patients^c^	112/564 (19.9%)	574/597 (96.1%)	4.84	4.10–5.72	157.8	72.8–342.1

CI, confidence interval; NA, not applicable due to convergence failures; ND, not determined; aOR, adjusted odds ratio; RR, risk ratio; URTI, upper respiratory tract infection

a Patients with intestinal parasitosis are mostly prescribed an antibiotic (metronidazole), yet this diagnosis cannot be given in the absence of microscopy and therefore is not included in ALMANACH. We therefore performed this analysis omitting pre-implementation parasitosis.

b All prescriptions lacking a concomitant diagnosis warranting antibiotic treatment (any bacterial infection) were considered inappropriate

c Patients from 6 months of age were eligible to receive Vitamin A supplementation. This applied to 564 and 574 patients observed pre- and post-implementation, respectively.

d Logistic regression models were adjusted for age, sex, climatic season (rainy or dry), facility, reported fever, clinically assessed fever, observer and IMCI training.

Before ALMANACH implementation, 135 (22.1%) patients were inappropriately diagnosed with intestinal parasitosis in the absence of microscopy, whereas no patients received this diagnosis after ALMANACH implementation. As this classification led to a prescription of antibiotics, we performed an additional analysis of overall antibiotic prescription excluding the children who were diagnosed with intestinal parasitosis. Though the diagnosis made a substantial contribution to antibiotic prescription, patients were still more than 3 times (RR = 0.31,95% CI = 0.25–0.38) less likely to receive an antibiotic treatment after the introduction of ALMANACH in the health facilities after excluding parasitosis-related antibiotic prescriptions.

Pre-ALMANACH implementation, antibiotic treatment was given to 116 (62.4%) of 186 children diagnosed with URTI and to 32 (9%) of 375 children diagnosed with URTI post-implementation, respectively. Out of the 116 antibiotic treatments for children being diagnosed with URTI pre-ALMANACH, 112 (96.6%) should not have resulted in an antibiotic prescription according to clinical guidelines whereas only one of the 32 antibiotic treatments in the post-implementation round was considered unreasonable. This translates into a RR of 0.03 (95% CI = 0.01–0.22), i.e. the risk of inappropriate antibiotic treatment for URTI is 30 times lower with ALMANACH than without. Concomitant diagnoses or classifications which indicated antibiotic prescription to be appropriate among children diagnosed with URTI included: acute ear infection (n = 2 pre-ALMANACH, n = 19 post-ALMANACH); skin infection (n = 2 pre-ALMANACH, n = 4 post-ALMANACH); possible streptococcal sore throat (n = 3 post-ALMANACH); burn (n = 3 post-ALMANACH); dysentery (n = 1 post-ALMANACH); and likely urinary tract infection (n = 1 post-ALMANACH).

With regard to prevention, among 1161 patients eligible for Vitamin A supplementation (≥ 6 months of age) the probability to be checked for Vitamin A was nearly 5 times greater with an RR = 4.84 (95% CI = 4.10–5.72) following ALMANACH implementation.

#### Other indicators of quality of care

**Figure 3:**
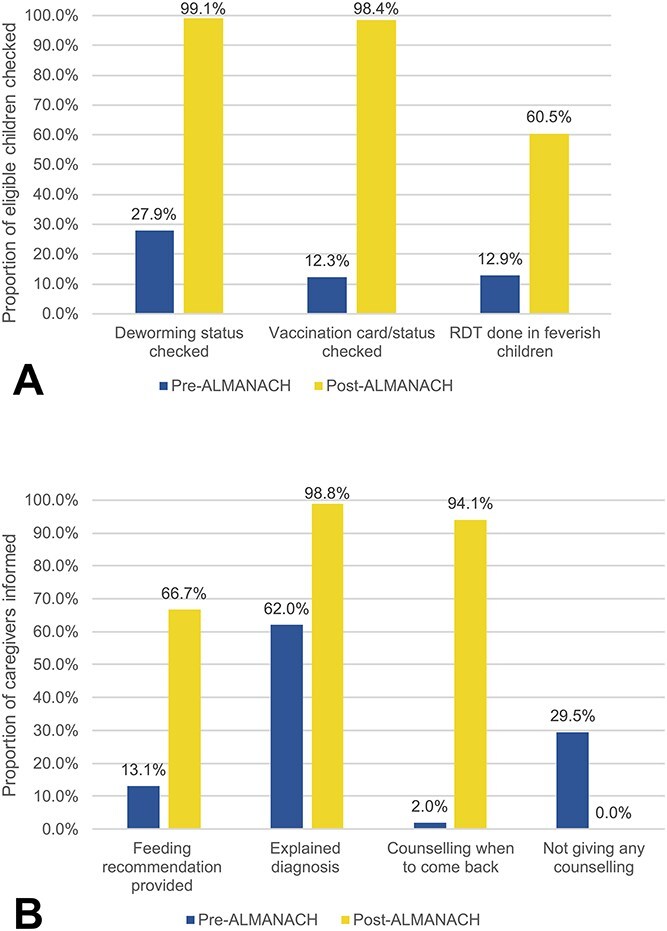
Indicators for quality of care assessed pre- and post-implementation. A: Proportion of eligible children benefitting from preventive and/or diagnostic measures pre- and post-ALMANACH implementation. All children were eligible for vaccinations, while 419 and 470 children pre- and post-ALMANACH, reached the age of 12 months to be given deworming medicines. Pre- and post-implementation 93 and 38 children, respectively, presented with fever. B: Proportion of caregivers of sick children who received health counselling before and after implementation of ALMANACH. In total 611 and 639 observations were analysed before and after implementation, respectively

Adherence to IMCI assessment was considered according to indication (i.e. some elements indicated for all children, and others depending on presenting symptoms and signs). General danger sign assessment, indicated for all children, was performed poorly prior to implementation of ALMANACH, with no child checked comprehensively for danger signs, and only eight (1.3%) checked for at least one danger sign. Post-ALMANACH, 634 (99.2%) of children were checked for all danger signs according to IMCI. Fever was checked or spontaneously reported for 343 children (56.1%) pre- and 629 (98.4%) post-ALMANACH. Of 93 febrile children pre-ALMANACH, malaria rapid diagnostic tests (RDTs) were performed on only 16 (12.9%) children with no reported stock-outs of tests (Fig. 3A); post-ALMANACH, 23 (60.5%) of the 38 febrile children had a malaria test and 15 (39.5%) were planned but not performed due to stock-outs. Additional RDTs were performed for history of fever or consumption of antipyretic medication (not shown as not comparable to the pre-intervention period). Respiratory rate measurement when indicated (with fever as per ALMANACH) was checked for nine (14.5%) children pre- and 20 (76.9%) children post-intervention. Skin pinch to assess dehydration was performed on children classified with diarrhea 37/122 (30.3%) pre- and 102/113 (90.3%) post-ALMANACH. Mid-upper arm circumference measures were available for 560 (99.5%) and 594 (99.5%) children 6 months of age and above pre- and post-ALMANACH, respectively. Pallor was checked for 44 (7.2%) and 622 (97.3%) patients pre- and post-ALMANACH, respectively.

ALMANACH also showed a beneficial effect on indicators of diagnosis, management and counselling (Fig. 3), other than those already presented within the primary and secondary outcomes. Caregivers were informed about the diagnosis of their child in 379 (62%) consultations pre- and 631 (98.8%) post-ALMANACH. No referrals were made during the pre-intervention period; 13 (2%) children were referred in the post-intervention period, including nine (1.4%) urgent referrals (three of which were refused by caregivers). Appropriate acute watery diarrhea (AWD) treatment was high—ORS was given to children with AWD 55/62 (88.7%) pre- and 107/112 (95.5%) post-ALMANACH, and zinc to 57/62 (91.9%) pre- and 104/112 (92.9%) post-ALMANACH.

ALMANACH also showed a beneficial effect on preventive measures other than Vitamin A. Deworming status was checked for eligible patients (12 months old and above) in 117/419 (27.9%) consultations pre- and 466/470 (99.1%) post-ALMANACH (Fig. 3A). Vaccination status was checked for 75 (12.3%) children pre- and 629 (98.4%) children post-ALMANACH.

Counselling on general feeding recommendations was low pre-ALMANACH (caregivers of 80 children, 13.1%) and improved but below full coverage post-ALMANACH (caregivers of 426 children, 66.7%). The most pronounced difference in counselling before and after implementation was seen for counselling on danger signs and advice on when children should be brought back to the health facility with 12 (2.0%) vs 601 (94.1%) of caregivers receiving the advice pre- and post-ALMANACH, respectively (Fig. 3B).

### Exit interviews with caregivers of sick children

Exit interviews were conducted with a total of 351 caregivers of sick children 2–59 months of age across seven facilities following implementation. ALMANACH was reported to have been used by 285 (81%) of caregivers; 26 (7%) were unsure if it had been used and the remained indicated it had not been used in the consultation. Out of the 285 caregivers who reported that it had been used, all (100%) were comfortable with ALMANACH being used, however only 104 (36%) were aware of it before the consultation. For those that were aware, 56 (54%) said it was because their child had been consulted with the tablet before, and 37 (36%) had heard about it through an information session at the facility with the remainder hearing from a relative or other personal contact or a community HW.

Almost all caregivers (327, 93%) reported having been informed of their child’s diagnosis by the HW and were able to report the diagnosis received. Medication was reported to have been given or prescribed to their child by 265 (75%) caregivers. Medication counselling was reported almost universally among those with medication or a prescription, with only 2 (1%) and 1 (<1%) out of 265 caregivers indicating that the medication had not been explained by the HW or did not feel comfortable with the prescribed drug, respectively. Only a minority of caregivers would have expected other treatments for their child (2%) while 231 out of 285 (81%) caregivers were satisfied with the treatment provided and 16% did not have any expectations during consultations with confirmed use of ALMANACH.

The majority of caregivers reported receiving health education while at the clinic (225, 64%), most of which was provided by the health promoter whilst awaiting consultation (163, 72%). Only 89 (25%) interviewees recalled having received health education in the community, and only 69 (20%) were aware of the community health committee attached to the facility.

Most respondents mentioned they only had to wait a little amount of time (214, 61%); 42 (12%) reported no waiting at all, but the remaining 27% perceived waiting times as long.

### Feedback from group discussions and engagement sessions

During the pre-implementation group discussions, both HWs and supervisors expressed concerns about potential dissatisfaction due to expected longer consultation time, mistrust of caregivers towards new technologies and feasibility of use of electronic devices in facilities lacking power supply. Most concerns raised before starting with ALMANACH have not materialized after the implementation and the HWs and supervisors continued to be highly motivated to use the application.

As a result of feedback from the pilot on the content of ALMANACH, adaptations to the CDA were undertaken including enhancements to classify and treat skin conditions and for the surveillance and management of malnutrition in view of an expected high prevalence of SAM [4]. Subsequently, changes were also made as a result of a change in medication availability and to align with new clinical guidelines (e.g. for the management of SAM). A total of five releases have been made, from the first version deployed in September 2020 to the latest release in September 2023.

Feedback from users and caregivers has consistently been very positive (Box 1), highlighting the role of ALMANACH in supporting improvements in quality of care and understanding of recommended evidence-based practices for sick children under 5 years of age.

**Box 1.** Qualitative data (i.e. quotes) on the acceptability of the ALMANACH tool from recipient and provider perspectives.

Recipient perspective (caregivers):

- *“For a long time I have been taking my child to a traditional healer whenever he is sick, and he applied concoctions on his chest which never helped my child. Since ALMANACH started, my child improved with the medications prescribed by the health worker“; Mother #1*
- *“I knew little about the importance of breastfeeding. When I came to the SRCS Clinic, the nurse encouraged me to read from the ALMANACH tablet at the end of the consultation where a counselling session is given, so now I read directly from the tablet and understood the messages“; Mother #2*

Provider and supervisor perspective:

- *‘The community is happy and appreciates ALMANACH. Equally, as a health worker, I feel supported by ALMANACH as it helps me to do my job better through guidance to conduct various on-site tests to confirm diagnosis. ALMANACH also reminds me to ask caretakers about prevention treatments, which was overlooked before ALMANACH. What makes me particularly excited are upper respiratory tract infections that I previously treated with antibiotics. Now these cases are given mere cough remedies and children improve a lot’; Health worker*
- *‘Beneficiaries have learnt the danger signs and severe complication of children. At triage we prioritize children with severe conditions and beneficiaries have accepted those children who require urgent attention to be prioritized’; Health promoter*
- *‘ALMANACH provided a local solution to the most common conditions: lemon, honey and tea were available ever since I was young but we did not know their effectiveness. With minor coughs we noted that antibiotics are not needed and we are teaching our communities the use of local remedies’; Community Health Committee member*
- *‘ALMANACH is a nice project and I am happy to implement ALMANACH in my clinics. Under 5 children are benefiting from ALMANACH by receiving good quality service and the community are appreciating ALMANACH for restricting misuse of drugs. In addition, the project improved the quality of consultations done by Under 5 nurses through proper history taking and physical examination as well as conducting rapid diagnostic tests to know the various diseases. ALMANACH has enhanced quality of care through prompting referral of eligible children to hospital for further treatment. I thank the project team for their support’¸ Supervisor SCRS Branch Health Officer*

## DISCUSSION

This evaluation demonstrated that the introduction of the ALMANACH IMCI-based CDSS was feasible and resulted in marked improvements in the quality of care, despite challenging circumstances of the health system and security context in South-Central Somalia. In addition, it enabled the identification of potential opportunities to further improve health services for sick children in the future.

Of note, during the time of the assessment, we did not see any major changes between the settings, which could easily explain some of these remarkable effects. Neither hindering seeking health care (e.g. a change in the burden of COVID, security, etc.), nor other influences (e.g. changes in staff) have been reported. For interpretation of results it has to be acknowledged that in evaluating ALMANACH, we did not distinguish between the instrument and the overall implementation package in terms of impact on quality of care, as it is accompanied by additional training and extensive monitoring, all of which contributed to the success of improving health care for children under 5 years of age in Somalia. However, given the high coverage of IMCI training and supervision pre-intervention, and the effect size demonstrated, it seems likely that impact was driven by the introduction of the digital tool.

The direct observations may have induced a Hawthorne effect, resulting in behavioral change whilst being observed [34]. However, as observations were structured and took place in both pre and post periods, with no healthcare provider staff turnover, we anticipate that any impact is unlikely to have modified the effect size. The possible exception to this assumption was that the uptake of ALMANACH was higher as a result of observation, which could generate a higher impact. Here too, however, we would only assume a minimal effect, as the uptake rate of ALMANACH was consistently high even without direct observations as indicated by routine data monitoring.

### Antibiotic use

The proportion of consultations ending with an antibiotic treatment was remarkably reduced from pre-ALMANACH to post-ALMANACH, even after adjusting for possible confounders. Overall antibiotic prescriptions reduced by more than 70%. This finding is comparable with results from Tanzania and Afghanistan where reduction in antibiotic treatments after ALMANACH implementation ranged between 78% and 86% [24, 28]. In Nigeria, however, the reduction in oral antibiotic prescriptions was much less pronounced with 10% at small-scale and 12% after large-scale implementation [23, 27]. For the large-scale assessment one reason may have been the lower baseline rate for these prescriptions that were at 34% [27]. In Somalia, the effect on unnecessary antibiotic treatment was even more striking if children diagnosed with URTI were considered. URTIs or other likely viral infections are one of the most common indications leading to unreasonable antibiotic prescriptions [28, 35]. After ALMANACH implementation in the study facilities only one single URTI diagnosed child (3.2%) was unreasonably treated with antibiotics compared to 96.6% prior to introduction of the tool. This demonstrated the utility of the tool in reducing unnecessary antibiotic prescriptions, avoiding potential adverse side effects for patients and mitigating emergence of antimicrobial resistance [36]. Fewer prescriptions of unnecessary medication (in our case mostly antibiotics) through more accurate diagnosis and guided treatment recommendations also helped to reduce costs and supports optimal use of existing resources [37]. In the case of health centers managed by the ICRC these costs were to be saved by the organization considering that medication was provided for free if in stock, and in other settings the saving might be on the patient side.

However, we have to put in perspective that our findings represent the prescription situation at day 0, while actual antibiotic prescriptions may still be higher taking into account potential follow-up visits [28], patient demand and easy access to antibiotics through pharmacies in rural Sub-Saharan Africa [38]. To a certain extent, the general monitoring of this situation was possible as HWs were requested to fill in any additional drug prescriptions or interventions at identified re-visits into a free-text field before ending the consultation with ALMANACH. This data was reviewed regularly and so far had not shown any major follow-up with antibiotic treatment where initially not recommended.

Treatment of URTI with unnecessary antibiotics decreased dramatically by a factor of 30, exceeding our expectations. Exit interviews suggested only 2% of caregivers would have expected other treatments for their child while 81% were satisfied with the treatment provided. This was a powerful finding as patient/caregiver expectation was often cited as a driver of antibiotic over prescription [39, 40]. Given that overuse of antibiotics is a major public health issue both for individual health and the rise of antibiotic resistance, both globally and in Somalia, this was a very encouraging finding to support the MoH of Somalia in antibiotic stewardship. The finding that ALMANACH improved antibiotic prescription was consistent with other studies [24, 28].

### Prevention

Major improvements could also be seen in the field of prevention and screening. The proportion of consultations in which vaccination status was checked went from 12% to 98%. The likelihood of being checked for the need of Vitamin A was nearly 5 times higher when ALMANACH was used. Checking for palm or eye pallor, a symptom of anemia, went from 7% to 97.3% of patients. Given the context of food insecurity, high malnutrition and high vaccine-preventable disease burden, these findings suggested use of ALMANACH improved opportunistic screening and prevention to reduce or address malnutrition in children and address immunization gaps. Finding improved prevention was consistent with other research on decision-support tools in low-resource settings [21, 23]. ALMANACH introduction also further enhanced appropriate management of diarrheal diseases with ORS and zinc, even though adherence levels to treatment guidelines for AWD were already high before.

### Additional benefits for quality of care

The use of ALMANACH allowed for additional identification of other foci for improvement of health care provision including training, quality of clinical examination and supervision. Similar to findings from CDSS evaluation studies in Afghanistan [24] and Burkina Faso [26], ALMANACH significantly improved adherence to physical examination tasks as highlighted by the increased rate of children checked for clinical danger signs, to identify the most critical patients for urgent referral and adequate pre-referral treatment, and clinical signs of anemia, vaccination and deworming status as well as the 5-fold higher number of feverish children that underwent a malaria RDT. Since most children were checked for palm or eye pallor, we did expect even many more children being identified with anemia and receiving appropriate treatment using ALMANACH. The most recent micronutrient survey from 2019 found 43.4% of children between 6 and 59 months of age anemic in Somalia [41]. Also methodologically ALMANACH offered potential for improvement of clinical assessments e.g. in checking for bilateral oedema or promoting more accurate measurements of clinical signs by specifying to count values (e.g. respiratory rate) exactly instead of just estimating them.

### Counselling and communication with caregiver

We found a substantial impact on counselling and communication with caregivers, consistent with findings from ALMANACH in Nigeria [27].

Every caregiver leaving the consultation conducted post-implementation got at least one type of health advice. This was further corroborated through the exit interviews confirming that all caregivers consulted with ALMANACH were able to state the diagnosis given to their child and was further underpinned by qualitative transcripts collected within sensitization and review meetings (see Box 1). The most striking differences were seen in providing health advice on when to come back with the child for follow-up including the explanations and guidance on urgent re-visits presenting any danger sign. In turn, this could result in impact beyond the primary care consultation in terms of improved clinical outcome seen in other studies [27]. The improved health counselling could further promote the communication, relationship and trust between the receiving communities and the providing health personnel.

### Community acceptance

These improvements in interaction may have a significant impact on the ongoing acceptance of ALMANACH by the community, which leads us to an important factor of success of the ALMANACH intervention. The continued engagement of the community, the caregivers, the supervisors as well as the HWs themselves seemed to be crucial to maintain the high level of acceptance and utilization of the tool. Exit interviews confirmed that all caregivers of sick children were comfortable with the new tool. 93% were aware of the diagnoses after their children was consulted using ALMANACH, indicating and further promoting an improved communication between healthcare workers and caregivers. 81% of caregivers also affirmed of having received a treatment as expected, which is surprisingly positive considering that ALMANACH notably reduced over-prescription of antibiotics as mentioned above.

Stakeholder engagement had an important role in the successful implementation of ALMANACH. This included focus group discussions with the end-users, continuous sensitization activities with the community and feedback collection from the caregivers. The implementing HWs had also repeatedly acknowledged its capacity to guide them particularly in the correct dosage of medicinal products. It has to be noted that major sensitization efforts were needed to maintain an understanding of ALMANACH in the communities. In particular, during the pilot implementation phase, many caregivers expressed frustration at not receiving antibiotic treatment or sometimes no medication at all.

An interesting finding was the median duration of the consultation, which went from 4 min and 18 s to 7 min and 12 s after ALMANACH implementation. The pre-implementation consultation time was shorter than anticipated to be needed for IMCI and may be indicative of insufficiently comprehensive care while increased duration may present a problem for resourcing [42–44]. While ¾ exit interview respondents said they did not have to wait or only very little, suggesting it is an acceptable duration in this context from the perspective of caregivers, it was an increase of almost 70% of consultation time, as a more thorough assessment (history taking and physical examination) did require more time. This increase could be problematic at larger scale or in more crowded facilities and would benefit from additional exploration to understand if the longer duration ended up disrupting the facility workflow, human resource needs etc. Based on regular feedback from the HWs, this did not seem to be a problem in our facilities up until today.

In the seven facilities assessed, 100% of the consultations post-implementation were conducted with the ALMANACH tablet. While this is remarkable and speaks to the hard work and commitment of all persons involved, it may not be representative of longer-term or larger-scale deployment as other studies showed lower uptake, which likely reduced the effectiveness of the intervention [45].

During the exit interviews a lower rate (81% confirmed, 11% unknown use) of the ALMANACH tablet use was recorded compared to our clinical observation assessments where all HWs used the device. This may be partly explained by adapted patient admission protocols by the MoH for severely malnourished children between the two rounds of exit interviews. This resulted in less children being eligible for consultation with ALMANACH. The interviews did not specify if the child was eligible or not. However, close monitoring of routine register data during supervision confirmed, that the utilization rate of ALMANACH was still nearly 100% for the eligible children.

### Limitations

One of the main limitations of our assessment is the nature of the pre-post design of the study. The observed changes may therefore be attributed theoretically in part or even wholly to secular trends. However, given the huge observed differences pre- and post-implementation, it was clear that ALMANACH had meaningful impact on all indicators. Given the very difficult operational space in Somalia, the focus was on accompanying the implementation rather than taking a research focus. Additionally, this assessment took place during the SARS CoV-2 pandemic, where direct access to patients and facilities was even more difficult. Future studies using experimental designs such as cluster randomized controlled trial designs could help address such contextual challenges to provide more rigorous evidence on the impact of the implementation of such digital tools on quality of care and health outcomes.

We were not able to study the effect of sensitization efforts in our context, yet it is likely that community engagement could better inform a CDSS implementation and ensure its sustainability in other similar settings [45]. Future CDSS evaluation studies should consider determining the potential of tailored information and social mobilization campaigns for the uptake and acceptability of the intervention. The difficult access and security situation of our study setting did not allow for re-assessment of the children seen at day 0. Consequently, we could not determine clinical outcomes such as cure rates, complications or clinical failure as well as if key messages on when to come back with the child were well understood by the caregivers.

Another aspect to be noted is that the PHC facilities were quite far from secondary health facilities. One of the aspects of good guideline adherence that should have been impacted by the use of ALMANACH was appropriate referral—which was, referring patients when needed, meaning they should access more advanced or specialized care, as well as not referring patients when not needed to avoid congestion in higher levels of care or unnecessary expense and trouble to the caregiver. Referrals were not assessed in our ALMANACH evaluation study, yet, it is a documented bottleneck in various settings that referrals may not be successfully completed, for a range of reasons such as no means of transport, no possibility to leave other family members or no financial means [46] and this could mitigate the impact of ALMANACH on health outcomes.

## CONCLUSION

Despite the very challenging context in South Central Somalia, we could show that the implementation of ALMANACH was not only feasible but also showed important improvements in quality of care for children 2 and 59 months of age. Most notable were the reduction of unnecessary antibiotics, improved screening and prevention and better communication with caregivers. These results supported the wider use of ALMANACH as a consultation decision support tool, along with training, supervision, maintenance, improvements and scope expansion, to reach and improve the healthcare for larger numbers of sick children under 5 years of age in Somalia.

## Supplementary Material

Table_S1_Catchment_population_of_the_study_facilities_oqae029

## Data Availability

The data underlying this article will be shared on reasonable request to the corresponding author and after consultation with the ICRC and SRCS.
