# Supplementary material for: Impact of digital clinical decision support on quality of care and antibiotic stewardship for children under five in South-Central Somalia
Source: Oxf Open Digit Health. 2024 Dec 2;2(Suppl 2):ii32–44. doi: 10.1093/oodh/oqae029 (PMC11936327; doi:10.1093/oodh/oqae029)
Supplement: Table_S1_Catchment_population_of_the_study_facilities_oqae029 [file Table_S1_Catchment_population_of_the_study_facilities_oqae029.docx]

**SUPPLEMENTARY MATERIAL**

**Impact of digital clinical decision support on quality of care and antibiotic stewardship for children under five in South-Central Somalia**

Eveline Hürlimann^1,3^, Marco Landi^4^, Alli Miikkulainen^4^, Camille Renner^2,3^, Capucine Musard^2,3^, Hassan Hussein Mohamed^4^, Hassan Abdullahi Ali^4^, Omar Sheik Mohamud^5^, Abdifatah Ahmed Mohamed^6^, Talia Salzmann^2,3^, Fenella Beynon^2,3^, Anja Junker^2,3^*

^1^Swiss Tropical and Public Health Institute (Swiss TPH), Department of Medical Parasitology and Infection Biology, Allschwil, Switzerland

^2^Swiss Tropical and Public Health Institute (Swiss TPH), Swiss Center for International Health, Allschwil, Switzerland

^3^University of Basel, Faculty of Science, Basel, Switzerland

^4^International Committee of the Red Cross (ICRC), Department of Primary Health Care, Somalia Delegation in Kenya (SOK), Nairobi, Kenya

^5^Somalia Red Crescent Society (SRCS), Department of Primary Health Care, Mogadishu, Somalia

^6^Ministry of Health & Human Service, Department of Primary Health Care, Federal Government of Somalia, Mogadishu, Somalia

*Correspondence: anja.junker@swisstph.ch

Anja Junker

Swiss Tropical and Public Health Institute, Swiss Center for International Health

Kreuzstrasse 2

4123 Allschwil

Switzerland

**Supplementary Material:**

- Table S1. Overall and under-five (U5) catchment population of the study

facilities

**Table S1.** Overall and under-five (U5) catchment population of the study facilities

| **Facility** | **Total catchment population** | **Catchment U5 population** |
| --- | --- | --- |
| Balat | 32213 | 6120 |
| Beletweyn | 21980 | 4176 |
| Dusamareb | 18987 | 3608 |
| Farjano | 18305 | 3478 |
| Galinsor | 18667 | 3547 |
| Hudur | 11022 | 2094 |
| Houlwadaag | 20922 | 3975 |
| Total | 142096 | 26998 |
